# The association between experiences of unfair treatment in school and adolescent adjustment over 5 years

**DOI:** 10.1111/jora.13023

**Published:** 2024-09-30

**Authors:** Eva Grew, Gülseli Baysu, Rhiannon N. Turner

**Affiliations:** ^1^ School of Psychology Queen's University Belfast Belfast UK

**Keywords:** adjustment, adolescents, discrimination, unfair treatment

## Abstract

This study investigates how high‐school experiences of unfair treatment connect to adolescents' higher education enrollment and life satisfaction 5 years later. We utilized four waves of data at ages 14 years (T1), 16 (T2), 18 (T3) and 19 (T4) in the UK (*N* = 13,065; 51% Male, 49% Female, 70% White, 11% Black, 19% South‐Asian). Perceived teacher unfairness at T1 predicted lower university aspirations (T2) and subsequently lower enrollment in higher education (at T3 and T4) and life satisfaction (T4). Experiences with T1 teacher ethnic discrimination also predicted lower life satisfaction. The effects were similar across historically minoritized and majority‐background pupils, but historically minoritized adolescents experienced more ethnic discrimination in school. Our findings highlight the importance of fair treatment for all pupils.

## INTRODUCTION

Unfair and discriminatory treatment in schools is unlawful in both the UK and the U.S. (Equal Educational Opportunities Act, 1974; The Equality Act, 2014) and should therefore no longer be a pressing issue in education. Yet, such treatment within schools prevails in subtle forms. A meta‐analysis by Tenenbaum and Ruck (2007) revealed that teachers held more positive expectations, made more positive referrals, and addressed pupils in positive (e.g., praise) or neutral speech (e.g., asking questions) comparatively more often for White majority versus historically minoritized Black pupils. Historically minoritized pupils may feel discouraged from taking advanced‐level classes more than their White peers and believe that they received lower grades than they deserved due to racial bias (Fisher et al., 2000). This is an issue not only because the topics of equity and justice should be vital in researching child development (Killen et al., 2016) but also because of the potential negative effect that the experiences of unfair treatment may have on students' school adjustment. For historically minoritized pupils, even supposedly subtle experiences of unfair treatment (“passive racism”) may be perceived as discrimination (Civitillo et al., 2023). However, it is also the case that not all experiences of unfairness in school are attributed to ethnic background, and perceived teacher unfairness is therefore also studied as a general student experience, irrespective of their ethnic background (e.g., Helm et al., 2020). In the present paper, we combine two lines of literature which are conceptually related but rarely studied together: general perceived teacher unfairness and ethnic discrimination. In doing so, we investigate the potential negative effect of these social experiences on adolescent adjustment in school among students from both historically minoritized and White ethnic backgrounds. Below we outline the findings from studies on both general teacher unfairness and ethnic discrimination, highlighting the role of ethnic background to better understand how unfair experiences in school impact academic aspirations, university enrollment, and socio‐emotional outcomes, particularly for historically minoritized students.

### Theoretical framework

We applied the ecological systems theory by Bronfenbrenner (1977, 1979) alongside García Coll et al.'s (1996) Integrative Model, and Tajfel and Turner's Social Identity Theory (Hogg, 2016) to understand how unfair treatment in school may affect adolescent adjustment. Growing evidence suggests that experiences of unfairness in school might be particularly detrimental for adolescents from certain ethnic groups (Benner et al., 2018; Civitillo et al., 2023). To examine why this is the case, we applied the ecological‐systems view of schools, according to which the proximal experiences (unfairness) and more distal layers of adolescent social ecology (racism and prejudice in the society) are interactively connected. Studies that target specifically historically minoritized adolescents often focus specifically on unfair treatment in schools attributed to *racial* unfairness or ethnic discrimination (e.g., Cooper et al., 2022; D'hondt et al., 2016). Such unfair experiences can make positive adjustment in schools difficult for historically minoritized pupils. We furthermore link this with the evidence that general teacher unfairness, studied irrespective of student ethnic background, was also shown to be detrimental for adolescent adjustment (Helm et al., 2020; Thomas et al., 2009). In their integrative model, García Coll et al. (1996) emphasize the importance of considering unfair experiences when studying the effects of social ecology on the development of adolescents from historically minoritized backgrounds, alongside more general constructs relevant to developmental processes among all adolescents. Specifically, they argue that adolescents from historically minoritized ethnic groups face challenging conditions within school, experiencing more discrimination and other types of negative relationships with peers and teachers, and these experiences uniquely affect their developmental processes.

Social identity theory may also help to explain the impact of unfair experiences on adolescent adjustment. Adolescents strive to develop a stable self‐concept through identification with their social groups (Hogg, 2016). Interactions that present a threat to social identity or group norms such as discrimination or unfairness (Baysu et al., 2016; Verkuyten et al., 2019) may result in adolescents from historically minoritized groups either detaching themselves from the perceived sources of threat—teachers, classmates, and eventually school in general—or by perceiving academic outcomes as less valuable. For adolescents from an African–American background, for example, perceptions of teacher unfairness are linked to lower academic achievement (Assari & Caldwell, 2018; Thomas et al., 2009) and lower school engagement (Griffin, 2018), and similar findings have emerged in studies with students from other minorities (Baysu et al., 2016, 2023). In line with predictions derived from these theoretical perspectives, below we provide an overview of research on how experiences of unfairness affect student adjustment, and specifically, whether these effects are moderated by adolescent ethnic background.

### Perceived unfairness in schools and school adjustment

Teacher unfairness is a term that has been researched under various labels including classroom justice (Chory‐Assad, 2002), perceived teacher fairness (Choi et al., 2019), or unfairness (Chen & Cui, 2020; Gini et al., 2018; Huang, 2020). These terms are based on the same premise; that in school, teachers are authorities with the power to distribute resources such as grades, attention, or support, and adolescents may sometimes feel that they are not receiving their fair share of these resources. Perceived teacher unfairness, the term we used in the present study, is understood not as a term contradictory to equality but instead as adolescents' perception that teachers treat them worse than others or “pick on them”, and it is associated with various negative academic and socio‐emotional outcomes for adolescents (Assari & Caldwell, 2018; Thomas et al., 2009). Unlike ethnic discrimination, which is explicitly related to one's social identity, teacher unfairness may be perceived as systematic or ad‐hoc.

A wealth of evidence suggests that various positive and negative relationships in the school microsystem are key to adolescent adjustment, as student–teacher or peer relationships in school are associated with academic and socio‐emotional outcomes (Chu et al., 2010; McGrath & Van Bergen, 2015; Polanin et al., 2021; Roorda et al., 2017). As such, perceived school unfairness may hinder adolescents' positive adjustment in school. School adjustment in the present study entails both academic and socio‐emotional outcomes that are crucial for adolescents' ability to adapt and thrive in a school environment, that is, being academically successful without compromising one's mental health. Focusing first on academic outcomes, young people who perceived their school or course as unfair were less engaged and motivated in it (Chory‐Assad, 2002; Ripski & Gregory, 2009), and teacher unfairness was associated with lower motivation and interest in school (Helm et al., 2020; Wentzel, 2002). Unfair treatment by teachers might also have negative effect on student achievement (e.g., Peter et al., 2012), with some mixed findings, suggesting its effects might be small (Chen & Cui, 2020) or even inverted (Ripski & Gregory, 2009).

Parallel to academic outcomes, perceived unfair treatment in school might also harm adolescents' socio‐emotional functioning, as perceived teacher unfairness is linked with psychological and somatic problems and lower satisfaction with friends and school (Gini et al., 2018). Students who experienced teacher unfairness also reported more anxiety about school and in turn lower life satisfaction (Huang, 2020). Conversely, being treated fairly by teachers may be a protective factor for mental health. In a nationally representative sample of South Korean adolescents, perceived teacher fairness buffered the negative effects of academic stress on life satisfaction (Choi et al., 2019). Furthermore, perceived fairness is also a characteristic of supportive teachers (Suldo et al., 2009), and supportive relationships with teachers predict positive socio‐emotional outcomes for adolescents (Cornelius‐White, 2007; Hoferichter et al., 2021). Positive school adjustment represents adolescents' ability to adapt and thrive in a school environment, that is, being academically successful without compromising one's mental health. As such, unfair treatment in school puts adolescent school adjustment at risk both through its effect on academic and socio‐emotional functioning.

While there is wealth of research on teacher unfairness, these studies do not generally focus on students' ethnic or racial background, despite the fact that (a) some students might be more likely to be treated more unfairly due to their ethnic background (Tenenbaum & Ruck, 2007) or (b) historically minoritized students may be more strongly affected by negative treatment in schools. In some cases, unfair treatment of historically minoritized adolescents in school might be labeled as discrimination (e.g., Assari & Caldwell, 2018), and perceived teacher unfairness and ethnic discrimination are traditionally parallel lines of research. Yet, topic of general teacher fairness is often examined in general population without considering student ethnic background. Teacher unfairness and school ethnic discrimination are both negative experiences interdependent within the microsystem, which are interactively linked with inequalities within the macrosystem, such as racism and prejudice towards certain ethnic groups (García Coll et al., 1996), with potential to affect the developmental processes (Bronfenbrenner, 1977, 1979).

### Ethnic discrimination and school adjustment

There is a substantial body of research on the negative effects of perceived ethnic discrimination from teachers and peers on academic outcomes among African–American adolescents (Cogburn et al., 2011; Eccles et al., 2006) and other minorities (Baysu et al., 2016; Benner et al., 2018; Civitillo et al., 2023). For instance, among historically minoritized adolescents in Belgium, D'hondt et al. (2016) found that frequent experiences of ethnic discrimination predicted a reduced sense of academic control, having potential negative implications for academic motivation. In a longitudinal study with Black adolescents, Cooper et al. (2022) found that experiences of ethnic school discrimination in school were associated with lower academic persistence and school satisfaction, and greater depressive symptoms 1 year later. Furthermore, historically minoritized adolescents are already at risk of having lower achievement than their White peers (Strand, 2010), and based on meta‐analytical findings, perceived ethnic discrimination predicts lower grades, less engagement and motivation in school, and worse socio‐emotional outcomes such as higher depression or internalizing symptoms (Benner et al., 2018; Civitillo et al., 2023; Cogburn et al., 2011). Discrimination may be particularly detrimental for adolescents in some ethnic groups, as its association with socioemotional distress might be stronger for adolescents of Asian descent (Benner et al., 2018) and its negative effect on well‐being might be particularly detrimental for African American students (Civitillo et al., 2023).

School discrimination may be perpetrated by both teachers and peers, because it does not stem from unequal positions defined by school hierarchy (teachers as authority over students) but rather from inequalities at the macro‐level. Students' classmates form a crucial part of the school microsystem (Bronfenbrenner, 1977), and the importance of peer relationships increases during adolescence (Li et al., 2011). Accordingly, negative cross‐ethnic peer interactions (e.g., peer victimization or discrimination) are linked with worse academic and socio‐emotional outcomes (Wang, 2021). Interconnected negative peer experiences like ethnic discrimination and ethnic peer victimization (Vitoroulis & Vaillancourt, 2015) are cross‐sectionally and longitudinally associated with worse socio‐emotional outcomes such as lower self‐esteem or life satisfaction and higher symptoms of depression among historically minoritized adolescents (Brenick et al., 2018; Greene et al., 2006; Huynh & Fuligni, 2010; Niwa et al., 2014). There is also some evidence that peer discrimination is associated with worse academic outcomes such as drop‐out (Peguero, 2011) or lower achievement (Huynh & Fuligni, 2010), although the findings are mixed. Other researchers (Benner & Wang, 2017) found links between peer ethnic discrimination and socio‐emotional, but not academic well‐being.

The above study also found an opposite trend for teacher perpetrated discrimination, aligning with a review of the research literature suggesting that teacher ethnic discrimination might be more important for academic outcomes than discrimination by peers (Verkuyten et al., 2019) and meta‐analytical findings that peer discrimination might have a stronger association with socio‐emotional outcomes than educator discrimination (Benner et al., 2022). Yet, the positive and negative qualities of student‐teacher and peer relationships are interactively linked (Endedijk et al., 2022) and both correlate with socio‐emotional and academic outcomes in the same (negative) direction (Benner et al., 2018, 2022). While both peers and teachers can be the perpetrators of discrimination within school, teachers have a strong position within the school hierarchy, and can manifest this power through fair, or unfair treatment of pupils in class. Such unfair treatment is not unique to just those from historically minoritized groups. But, as we argue below, further research is needed to establish how adolescents specifically from ethnically minoritized groups are affected by experiences of unfairness, including general teacher unfairness and perceived ethnic discrimination exhibited by teachers or peers.

Despite the parallel between the negative effects of teacher unfairness and school discrimination on student adjustment, few studies have researched these two concepts simultaneously. In a rare exception, Benner and Graham (2011) longitudinally measured teacher discrimination and school fairness (as a part of school climate), concluding that discrimination predicted worse school outcomes through school climate. To our knowledge, however, no study has simultaneously researched the effects of adolescents' experiences of general teacher unfairness and ethnic school discrimination on academic and socio‐emotional outcomes, and most of the presented evidence is correlational, based on cross‐sectional studies.

### Role of ethnic background for the experiences of unfairness and school adjustment

Based on the evidence reviewed, it is clear that unfair experiences in school from teachers and classmates like unfairness or discrimination are negatively linked to students' adjustment in school. Yet we do not fully understand the dynamics which translate immediate relationships into real‐life longitudinal outcomes, in particular for adolescents from historically minoritized backgrounds. On the one hand, by focusing solely on ethnic discrimination among students from historically minoritized ethnic groups, we may miss the opportunity to learn about other, more subtle experiences of unfair treatment in school, or instances which were not perceived as ethnically motivated by the responding adolescent. Moreover, studies with historically minoritized students have sometimes failed to explicitly distinguish whether the experiences of unfair treatment were attributed to ethnic or religious reasons (Assari & Caldwell, 2018) or specify the perpetrator (Benner et al., 2018). On the other hand, studies focusing on generic teacher unfairness generally do not take into consideration ethnic background as one of the crucial factors linked to inequalities in school context (e.g., Chen & Cui, 2020). Based on the theories and literature reviewed above, we propose mediation and moderation models to investigate the role of macro‐level contextual factors in adjustment among historically minoritized and majority adolescents in the UK.

#### Mediation model

From the ecological systems view macrosystem influences such as prejudice and racism may trickle down into school microsystem (García Coll et al., 1996), affecting adolescents' social experiences and, through these experiences, their developmental outcomes; as such, historically minoritized adolescents might be particularly at risk of being exposed to unfair and discriminatory treatment. We therefore theorize that historically minoritized pupils might experience more negative interactions in schools, which may mediate the path between ethnic group and developmental outcomes. For example, historically minoritized pupils are at higher risk of racist peer‐victimization than majority (White) adolescents (Huynh & Fuligni, 2010; Verkuyten & Thijs, 2002), and adolescents from visible minorities experience more educational discrimination and teacher unfairness (Fisher et al., 2000; Tenenbaum & Ruck, 2007). Accordingly, we suggest that historically minoritized adolescents might experience more unfair treatment in school which in turn may explain (partially) their disadvantages in academic and socio‐emotional adjustment.

#### Moderation model

The mediation model discussed above does not necessarily explain the findings that adolescents from certain minorities are at higher risk for negative adjustment outcomes; such as that Black or Latinx adolescents exposed to violence at school are at particular risk of negative academic consequences such as dropping out (Peguero, 2011), or that the link between discrimination and psychosocial outcomes (socioemotional distress, well‐being) was stronger for adolescents in certain ethnic groups than others (Benner et al., 2018; Civitillo et al., 2023). We theorize that this is due to the threat such interactions present to social identity. The experiences of unfair treatment, in particular discrimination, could threaten the social identity needs of adolescents from historically minoritized ethnic groups (Verkuyten et al., 2019). Accordingly, we suggest a second (potentially) competing theoretical model to examine the processes framing historically minoritized adolescents' experiences of unfairness in secondary school; a moderation model. In line with this model, youth from historically minoritized groups feel less included and perform worse in schools when their ethnic identities are threatened (Baysu et al., 2016, 2023).

### Current study

In the current study, we sought to answer the following questions: Do experiences of unfairness and discrimination in postprimary education predict adolescent school adjustment later in life, defined in terms of academic aspirations and mental health problems in middle adolescence, and university enrollment and life satisfaction in late adolescence (RQ1)? Is ethnic group a moderator of the effects of unfair school relationships on school adjustment (moderation model), or is ethnic group a predictor of more negative school relationships, which in turn mediate the effects on school adjustment (mediation model) (RQ2)?

To achieve this, we used a large nationally representative sample of UK adolescents who were followed over 5 years to research the long‐term effects of adolescents' peer and teacher school relationships on their academic (higher education entry) and socio‐emotional (life satisfaction) outcomes over 5 years. Longitudinal evidence shows that relationships in the early years of postprimary education may influence distal outcomes such as students' academic aspirations and adolescent academic choices (Grew et al., 2022), and that perceived school discrimination may influence Black adolescents' education aspirations, greater depressive symptoms and lower school satisfaction (Cooper et al., 2022). We therefore aim to investigate whether school unfair experience (from both teachers and peers) would be linked with later enrollment in higher education (HE) and life satisfaction through the change (increase or decrease) in academic aspirations and mental health problems in secondary school. We base our research around a large and nationally representative sample of adolescents living in England, answering the call for more longitudinal research with large and diverse samples to research the topics of equality in education (Killen et al., 2016) and for studies outside of the U.S. (Civitillo et al., 2023), which presently dominate the social psychological research on ethnic discrimination (Benner et al., 2018; Zick et al., 2008).

**FIGURE 1:**
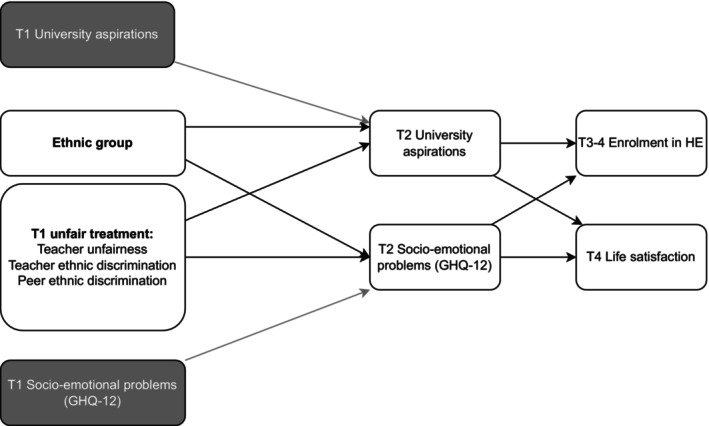
Structure of the main effects model. Control variables adolescent sex, household salary, and parental education included in all models.

Our predicted model is shown in Figure 1. We hypothesized (H1) that adolescents' experiences of school unfair treatment (teacher unfairness, teacher and peer ethnic discrimination) during postprimary years could harm their life satisfaction and the likelihood to attend higher education. We also explored the processes through which negative school experiences affect adolescent outcomes. Given that adolescents' developmental outcomes are interconnected, we explored whether the effects of school unfairness on HE enrollment and life satisfaction will be mediated via university aspirations and/or via socio‐emotional problems in further education. While the previous longitudinal evidence is scarce, based on some previous studies (e.g., Grew et al., 2022) we hypothesize (H2) that experiences of unfairness would be associated with lower aspirations and greater socio‐emotional problems, and in turn lower life satisfaction and HE enrollment. Finally, we applied an exploratory approach to learn whether either of our proposed models (moderation expectation vs. mediation expectation) reflects the processes connecting ethnic background to school unfairness and adjustment. Thus, we investigated whether historically minoritized adolescents experience more unfair treatment in schools (and in turn worse adjustment) or whether they are affected by such instances differently than their peers from White ethnic group.

## METHODS

### Participants

The sample was drawn from a nationally representative longitudinal study of young people in England (LSYPE, now called Next Steps) which conducts annual surveys during different school years (University College London, 2021). We utilized four time points of data collected between 2005 and 2010 following adolescents from ages 14 years (time 1), through to 16 (T2), 18 (T3) and 19 (T4). The ages at each time point are approximate, as the recruitment process took place via approaching adolescents in specific school years, meaning that some adolescents could have been slightly younger at the time when data collection took place, depending on their date of birth. The data we use in the present study spread from the second year of postprimary education until young adulthood; in England, postprimary education starts around the age of 11, and after the age of 16, students may continue into further (upper secondary) education, and eventually enter higher education around the age of 18. Out of 13,539 adolescents surveyed at T1, we analyzed those who could be assigned the White, Black, or Asian (or South‐Asian) ethnic group membership. To clarify, in the Asian group, 89.7% of adolescents in this aggregated group came from South‐Asian backgrounds. The rest of the adolescents included in this group self‐described their background as White and Asian or “any other Asian background” not specified above. We used the term “South‐Asian” to distinguish from findings about adolescents from Asian background in the U.S. literature, as the “Asian” group in the U.S. often refers to adolescents from East‐Asian (e.g., Chinese) backgrounds. In the final sample (*N* = 13,065; Male: 51%; Female: 49%), most adolescents self‐identified as White (70%; mainly White‐British). We aggregated the historically minoritized groups into two: Black (11%; mainly African, Caribbean, or mixed) and South‐Asian (19%; mainly from Indian, Pakistani or Bangladeshi background), considering mixed‐background pupils as minorities (Campion, 2019). We applied weights, which affected the size of ethnic groups in our analyses. See Data S1 for detailed ethnic compositions of each group. At T1 and T2, adolescents and their parents were surveyed face‐to‐face in their home. T3 and T4 survey involved an interview with the young person only, conducted either online, over the telephone or face‐to‐face (Department for Education, 2011). As this was a secondary analysis, we used all data available, and given the large sample size, it is reasonable to assume that we can detect even very small effects. This was supported by a post‐hoc power analysis in G*Power (Faul et al., 2007) for nine predictors which showed that the estimated power of our study was 1.00 under our expectation of small population effects (*f* ^2^ = 0.02, at α = .01).

### Measures

For detailed information about the items, see the technical reports from T1 (LSYPE W2 documentation), and T2 (W4). T3 (W6) and T4 (W7), which can be downloaded alongside the dataset. See Data S1 for details on the specifics of how the items used in our analyses were recoded from the original items in the dataset University College London (2021).

*Teacher (un)fairness (T1*) was calculated as the mean of six self‐reported items, recoded as binary (0 = *absence of unfair treatment*, 1 = *experienced unfair treatment*). The original scoring on these six items had to be adapted in our study to allow us to combine items measured on different response scales. One originally a 5‐point item “I get treated unfairly by my teachers” was recoded as 0 = *none*, 1 = *hardly any* to *all teachers*. The remaining five items which asked about experiences like being unfairly graded or disciplined (originally on 3‐point response scale) were recoded such as “If I get caught breaking school rules then usually I'm:” (0 = *less likely or the same to be punished*, 1 = *more likely to be punished*). As these items were not a standardized measure, we established the scale validity via confirmatory factor analysis using a robust maximum likelihood (MLR) estimator. All items had strong factor loadings (λ > .5). While there is no suitable method for estimating the reliability of binary item scales in complex studies with an MLR estimator, applying (Raykov et al., 2010) approach with an ML estimator, the scale is reliable, ρ = 0.78. Our decision to recode these items was also justified as we focus on the presence (vs. absence) of different types of unfair treatment, as for example if “hardly any” teachers treat adolescents unfairly, this could still be a negative experience if it is persistent.

*Teacher ethnic discrimination (T1)* represents if the adolescent responded positively to these yes or no items: “Do you think you were ever treated unfairly by teachers at your school because of (1) your skin color or ethnic origin?” (2) “your religion?” (0 = *no* [to both items], 1 = *yes*).

*Peer discrimination (T1)* was also measured with one binary (0 = *no*, 1 = *yes*) variable measuring if the adolescent reported that they were peer‐victimized because of racist motives.

*Socio‐emotional problems (T1, T2)* were measured via the General Health Questionnaire GHQ‐12 (Goldberg & Williams, 1988). Originally designed as a screening device identifying minor psychiatric disorders, this scale can be used for assessing the inability to carry out normal functions and experienced distress among adolescents (GL Assessment, n.d.). We calculated the mean of all 12 items, each scored on a 4‐point scale, with higher values indicating worse socio‐emotional functioning. The psychometric qualities of the GHQ‐12 were comparable at T1 and T2, see Data S1 (Figures S1 and S2) for details on longitudinal measurement invariance testing.

*University aspirations (T1, T2)* were measured with one item: “How likely do you think it is that you will ever apply to go to university to do a degree?” scored on a 4‐point scale (1 = *not at all likely*, 4 = *very likely*). This item was used as a measure of aspirations (Croll & Attwood, 2013) and, combined with other items (including “How likely do you think it is that if you do apply to go to university you will get in?”) as a measure of educational expectations (Khattab, 2015; Lazarus & Khattab, 2018). We used the single item as it is reflective more of adolescents' aspirations than their expectations.

*Higher Education enrollment (T3, T4)* was a binary variable, indicating whether the young person was (1) or was not (0) attending a Higher Education (HE) institution.

*Life satisfaction (T4)* was measured with the following item: “How dissatisfied or satisfied you are about the way your life has turned out so far?” (1 = *very dissatisfied*; 5 = *very satisfied*). Single‐item measures were shown as suitable for measuring life satisfaction, comparable with multiple‐item measures (Cheung & Lucas, 2014).

*Control variables (T1)* were gender (0 = *Male*, 1 = *Female*), household income (in £10,000), and parental education (1 = *no qualification*, 7 = *Degree or equivalent*).

### Analysis plan

The analyses were conducted in Mplus 8.3 (Muthén & Muthén, 2019) using MLR estimator with Monte Carlo integration. T1 weights were applied in all analyses and as instructed in the LSYPE user guide, a complex‐type analysis with clustering and stratification was used. We included the covariances among all T1 predictors and control variables as it was reasonable to assume these would be related. Missing data were estimated using maximum likelihood estimation. We centered all continuous predictors for easier interpretation. For analysis, we first tested the main effects of all T1 variables on school adjustment (see Figure 1). We measured school adjustment both in terms of academic outcomes (university aspirations and actual HE enrollment) and socio‐emotional outcomes (socio‐emotional problems and life satisfaction). We expected that more proximal outcomes would be associated with more distal outcomes, such that an increase in T2 university aspirations and a decrease in T2 socio‐emotional problems (controlling for T1 outcomes) would be associated with a higher likelihood of HE enrollment and higher life satisfaction at T3 or T4. Thus, T2 outcomes would function as mediators connecting T1 unfair treatment and ethnicity with adolescents' outcomes at T3–4. Then, we tested the processes through which adolescents' ethnicity was related to their school adjustment in two competing models: in the moderation model, we included the interactions between each predictor and ethnicity; in the mediation model, ethnic group was included as a predictor of T1 unfair treatment and discrimination; the rest was similar to the main effects model. Since in large samples, even tiny effects are detectable, we only considered effects at *p* < .01 as statistically significant.

## RESULTS

Descriptive statistics are presented in Table 1. The significant correlations between our study variables showed that concurrently, perceived unfair and discriminatory treatment in secondary schools by both peers and teachers were associated with worse academic (lower aspirations at T1) and socio‐emotional outcomes (more socio‐emotional problems at T1).

**TABLE 1:** Means, SD, and correlations for continuous variables, proportions for binary variables.

	*M*	SD	1	2	3	4	5	6	7	8	9	10	11	12	13
Continuous variables															
1. T1 University aspirations	2.77	1.07													
2. T2 University aspirations	2.73	1.22	.66***												
3. T1 GHQ‐12	0.85	0.51	.03**	.04***											
4. T2 GHQ‐12	0.86	0.49	.13***	.11***	.43***										
5. T4 Life satisfaction	3.99	0.91	.10***	.14***	−.14***	−.19***									
6. T1 Teacher unfairness	0.24	0.27	−.23***	−.23***	.17***	.07***	−.11***								
7. Parental education	4.59	1.84	.29***	.32***	.05***	.09***	.09***	−.11***							
8. HH salary	3.64	3.12	.24***	.25***	.01	.03*	.12***	−.10***	.43***						
Binary variables															
9. Asian	19%		.15***	.16***	−.03**	.01	−.02	−.03***	−.18***	−.10***					
10. Black	11%		.08***	.09***	−.01	.01	−.02*	.07***	−.02	−.06***	−.06***				
11. T1 Peer discrimination	5%		−.04**	−.04***	.08***	.03*	−.02	.12***	−.05***	−.05***	.11***	.07***			
12. T1 Teacher discrimination	16%		−.05***	−.05***	.05***	.04**	−.03*	.19***	−.11***	−.07***	.13***	.18***	.21***		
13. Sex (female)	49%		.12***	.13***	.23***	.22***	.04**	−.10***	.01	.01	.01	.01	−.05***	−.06***	
14. T3 and T4 HE enrollment	49%		.52***	.67***	.04**	.09***	.19***	−.23***	.33***	.26***	.11***	.03**	−.06***	−.08***	.09***

*p < .05; **p < .01; ***p < .001.

### The effects of unfair treatment and ethnicity in the main effects model

**FIGURE 2:**
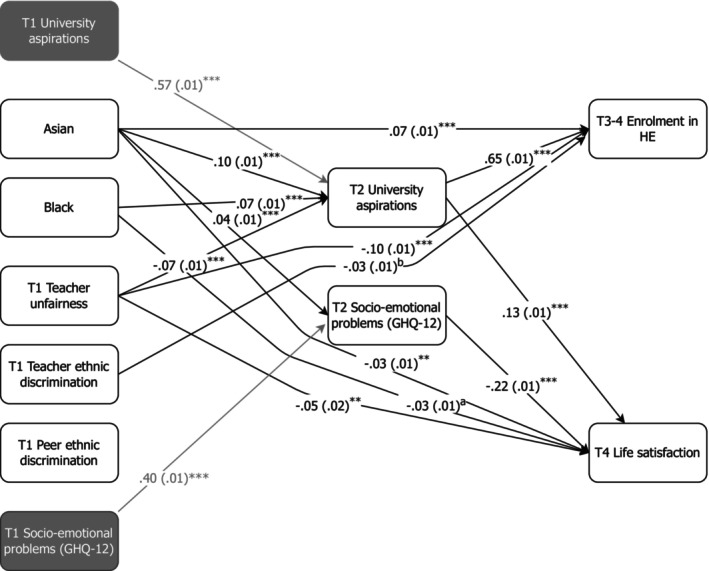
Main effects as standardized regression coefficients with standard errors in parentheses. **p < .01; ***p < .001; ^a^ *p* = .011; ^b^ *p* = .015. Only significant (*p* < .01) effects shown. Effects of control variables sex, household salary, and parental education at T1 not shown in the figure.

The main effects are presented in Table 2, and Figure 2, and the indirect effects of T1 predictors on T3–4 outcomes via T2, are shown in Table 3. As we used two mediator variables at T2 (university aspirations and socio‐emotional outcomes), we report the total and specific indirect effects. For example, if only the specific indirect effect via T2 aspirations was significant, this would indicate that aspirations were the sole mediator.

**TABLE 2:** Main effects of T1 variables with standard errors, and odds ratio for HE enrollment as binary outcome.

	T2 university aspirations			T2 GHQ‐12			T3–4 enrollment in HE				T4 life satisfaction		
	*b*	SE	*p*	*b*	SE	*p*	*b*	SE	*p*	OR	*b*	SE	*p*
T1 predictors													
Asian	0.50	0.03	<.001	0.07	0.02	<.001	0.74	0.10	<.001	2.10	−0.10	0.04	.008
Black	0.40	0.04	<.001	0.01	0.02	.461	−0.05	0.12	.665	0.95	−0.11	0.04	.011
Teacher unfair treatment	−0.33	0.04	<.001	0.04	0.02	.058	−1.03	0.14	<.001	0.36	−0.17	0.05	.001
Teacher discrimination	−0.05	0.03	.116	0.04	0.02	.030	−0.24	0.10	.015	0.79	0.03	0.04	.522
Peer discrimination	−0.10	0.05	.075	−0.03	0.03	.356	−0.32	0.17	.053	0.72	0.04	0.06	.479
T1 controls													
Parental education	0.09	0.01	<.001	0.02	0.00	<.001	0.22	0.02	<.001	1.24	0.01	0.01	.146
HH salary	0.02	0.01	<.001	0.00	0.00	.694	0.08	0.02	<.001	1.09	0.02	0.01	<.001
Sex	0.12	0.02	<.001	0.13	0.01	<.001	−0.04	0.07	.559	0.96	0.12	0.02	<.001
T1 outcomes													
T1 University aspirations	0.65	0.01	<.001										
T1 GHQ‐12				0.38	0.01	<.001							
T2 outcomes (as mediators):													
T2 GHQ‐12							0.16	0.07	.024	1.17	−0.40	0.03	<.001
T2 University aspirations							1.53	0.04	<.001	4.59	0.09	0.01	<.001
*R* ^2^	.48			.21			.61				.08		

**TABLE 3:** Standardized indirect effects (IEs) of T1 unfair treatment and ethnicity on T3 and T4 outcomes through T2 outcomes.

Outcomes	T3–4 enrollment in HE			T4 life satisfaction		
	Total IE	Specific IEs		Total IE	Specific IEs	
T1 predictors	β	via T2 Uni. Aspirations	via T2 GHQ‐12	β	via T2 Uni. Aspirations	via T2 GHQ‐12
Asian	.07***	0.07***	0.00*	.01*	0.01***	−0.01***
Black	.05***	0.05***	0.00	.01**	0.01***	−0.00
Teacher unfairness	−.05***	−0.05***	0.00	−.01***	−0.01***	−0.01
Teacher discrimination	−.01	−0.01	0.00	−.01**	−0.00	−0.01*
Peer discrimination	−.01	−0.01	0.00	.00	−0.00	0.00

*p < .05; **p < .01; ***p < .001.

Investigating our first research question (RQ1), unfair treatment by teachers in secondary schools (T1) had a statistically significant effects on school adjustment. Specifically, experiences of T1 teacher unfairness predicted lower university aspirations at T2, and a lower likelihood of attending HE at T3 or T4 (both directly and indirectly, through decreased aspirations at T2) as well as lower life satisfaction at T4 (both directly and indirectly, through decreased aspirations at T2). Those who reported that they were discriminated against by teachers due to their ethnic or religious background at T1 also directly had a lower likelihood of attending HE at T3 or T4. We did not find any significant effects of peer‐perpetrated T1 ethnic discrimination over time (at T2, T3, or T4).

Adolescent ethnic background predicted school adjustment at T2, and sequentially T3 and T4, both directly and indirectly. Controlling for T1 aspirations and socio‐emotional problems, adolescents from the South‐Asian group reported increased university aspirations but also increased socio‐emotional problems at T2 compared with their peers from the ethnic majority (White). Moreover, those from the South‐Asian group were more likely to attend HE at T3–4 (both directly and indirectly, through T2 university aspirations), while simultaneously reporting lower satisfaction with life at T4 (directly). We also found some evidence of small indirect effects from South‐Asian ethnicity to life satisfaction–a positive indirect effect through increased T2 aspiration and a negative indirect effect through increased T2 socio‐emotional problems, however when combined (as total indirect effect), these effects counterbalanced each other and no longer reached significance at *p* < .01. Similarly, adolescents from the Black group reported increased T2 aspirations, and in turn were more likely to attend HE at T3–4, fully mediated via T2 university aspirations. They also experienced lower life satisfaction at T4, yet this negative direct effect was diminished by a positive indirect effect, where their higher aspirations at T2 led to slightly better life satisfaction at T4. Overall, these findings suggest that compared with adolescents from the majority (White) group, historically minoritized pupils had better academic outcomes yet worse socio‐emotional outcomes.

### Do effects differ across ethnic groups? Moderation vs. mediation models

**FIGURE 3:**
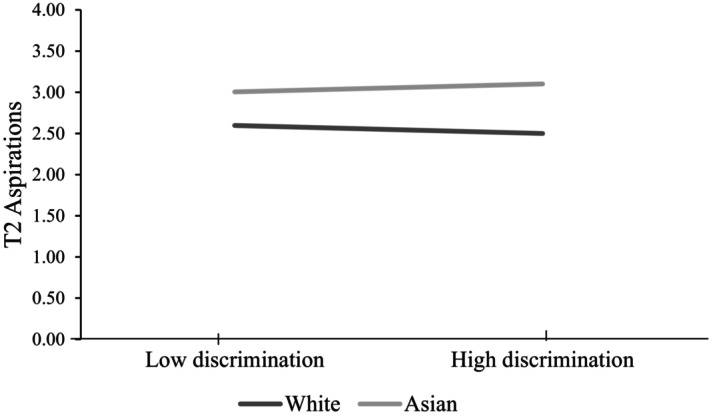
T2 university aspirations conditional on ethnicity and perceived teacher discrimination at T1. Differences between aspirations at low versus high discrimination tested via Wald test not significant in the White (*p* = .019) or the Asian group (*p* = .059).

We then investigate our second research question (RQ2). To test the moderation by ethnicity, we added six interaction terms (2_ethnic groups_ × 3_types of unfair treatment_) to the main effects model. Adding the interactions into the main effects model did not add any additional variance in the outcomes. We found only one interaction significant at *p* < .01 between teacher discrimination and South‐Asian ethnic background, *b* = 0.19, SE = 0.07, *p* = .004 (Table S3 for full results). Accordingly, adolescents from the South‐Asian group who experienced teacher discrimination at T1 surprisingly reported a slight increase in their aspirations at T2 (see Figure 3). However, the magnitude of this interaction was very small, accounting only for a small increase in aspirations from 3.01 to 3.10 (aspiration could range from 1 to 4), and the difference in aspirations of South‐Asian adolescents who did versus did not experience teacher discrimination at T1 was not statistically significant, Wald test *p* = .059. Overall, we did not find strong evidence for our moderation hypothesis.

We then tested whether the effects of ethnic background on school adjustment were mediated through experiences of unfairness in secondary school. Overall, pupils from historically minoritized ethnic backgrounds experienced more unfairness and discrimination in school than their majority‐background peers. Adolescents in both historically minoritized groups were more likely to report peer and teacher ethnic discrimination at T1. Specifically, those in the South‐Asian group were about four and three times more likely to report peer and teacher discrimination respectively (*b* = 1.33, SE = 0.13, *p* < .001, Odds Ratio (OR) = 3.77; *b* = 1.14, SE = 0.08, *p* < .001, OR = 3.13). Those in the Black group were three and six times more likely to report peer and teacher discrimination respectively (*b* = 1.20, SE = 0.15, *p* < .001, OR = 3.32; *b* = 1.75, SE = 0.09, *p* < .001, OR = 5.74). Furthermore, adolescents in the Black group reported slightly more teacher unfairness (which was not specifically related to ethnicity or religion) at T1 (*b* = 0.07, SE = 0.01, *p* < .001). In contrast, adolescents from the South‐Asian group reported slightly less teacher unfairness (*b* = −0.05, SE = 0.01, *p* < .001). Subsequently, T1 experiences of unfairness from teachers (but not peers) and ethnicity were directly related to later school adjustment (i.e., T2, T3, orT4 academic and socio‐emotional outcomes); these effects were comparable with those found in the main effects model (Table S4 for full results). We also tested the *indirect* effects of ethnic backgrounds on T2, T3, andT4 outcomes via T1 teacher ethnic discrimination, T1 teacher unfairness, and T1 ethnic peer discrimination. To calculate the indirect effects, teacher and peer ethnic discrimination variables were considered as continuous as testing the indirect effects with MLR estimator and logit regression coefficient is not possible in Mplus, and some authors argue that binary mediators can be treated as continuous (Gomila, 2021). These indirect effects, presented in Table 4, suggest that while a mediation between ethnic background and school adjustment might occur through teacher unfairness at T1, these effects are very small. Overall, while there was some evidence for the mediation hypothesis, more research is needed.

**TABLE 4:** Standardized indirect effects (IEs) of ethnicity.

	Total indirect effect (IE)	Specific IEs		
		Via T1 peer ethnic discrimination	Via T1 teacher ethnic discrimination	Via T1 teacher unfairness
	β	β	β	β
Indirect effects on T2 outcomes				
T2 Uni. Aspirations				
Asian	.000	−.002	−.002	.003***
Black	−.008***	−.001	−.002	−.004***
T2 Socio‐emotional problems (GHQ‐12)				
Asian	.001	−.001	.003*	−.001
Black	.005*	−.001	.004*	.001
Indirect effects on T3 and T4 outcomes^a^				
T3–4 Enrollment in HE				
Asian	.068***	−.002	−.003*	.004***
Black	.029***	−.002	−.005*	−.006***
T4 Life satisfaction				
Asian	.010**	.001	.001	.002**
Black	.006	.001	.002	−.003***

^a^ For T3 and T4 outcomes, the total indirect effects consist of a total of 11 specific indirect effects via (a) T1 unfair treatment and discrimination (3 specific IEs reported in the table), (b) via T2 outcomes (2 specific IEs) and (c) via their combinations (6 IEs, e.g., specific IE of T1 teacher unfairness + aspirations). The specific effects via T2 outcomes are omitted from the table for simplicity.

*p < .05; **p < .01; ***p < .001.

## DISCUSSION

In the present study, we address the need for longitudinal studies researching the effects of unfair treatment among majority, and in particular historically minoritized students outside of the U.S. context (Civitillo et al., 2023). We utilized a nationally representative and impressively large (over 13,000 adolescents) secondary dataset based on a longitudinal study of UK adolescents to develop our understanding of how proximal school relationships affect adolescents' lives through the context of un/justice and un/fairness embodied within the culture. As such, we have focused our study on adolescents from historically minoritized ethnic backgrounds, who might be at risk of being marginalized within the majority‐centered society (Tenenbaum & Ruck, 2007), yet we were able to compare their experiences with those of their peers who come from the majority, White background.

According to ecological systems theory (Bronfenbrenner, 1977, 1979), peer and teacher relationships are central to adolescent development, and negative, unfair and discriminatory proximal experiences might in turn be linked to more distal mental health and school outcomes (Benner & Graham, 2011; Cooper et al., 2022; Polanin et al., 2021). These academic and socio‐emotional outcomes shape the future opportunities of adolescents and could therefore help to close or widen the inequalities between adolescents from different backgrounds (Strand, 2010). In our study, we thus researched the effects of unfair treatment in secondary schools on university aspirations and socio‐emotional problems in further education, and in turn on access to higher education and life satisfaction in late adolescence/early adulthood. We found that adolescents' experiences with unfair and discriminatory treatment in postprimary education were associated with later negative socio‐emotional and academic outcomes spreading from middle to late adolescence.

We included two types of unfair treatment. The first represents general school unfairness that stems from teachers' position of authority with the power to act un/fairly when distributing resources, such as when adolescents feel like their teachers do not give them as much praise or are more likely to punish them compared with other students. The second type of unfair treatment, perceived discrimination, is related to adolescents' social identity, feeling that their ethnic, racial, or religious background is the reason why other people in school treat them negatively. Both peers and teachers can be perpetrators of discrimination, as its origins not from the power imbalance due to predefined formal roles within school (teacher vs. student) but rather from the unequal social status of groups within society (García Coll et al., 1996). We used data from four different time points, following the lives of UK adolescents from various ethnic backgrounds over 5 years. This allowed us to investigate the processes through which historically minoritized backgrounds might be linked to adolescent adjustment during postprimary years while addressing the lack of longitudinal studies with diverse samples (Killen et al., 2016), particularly outside of the U.S. context (Benner et al., 2018; Civitillo et al., 2023; Zick et al., 2008).

### Unfair experiences in school

Our findings from the longitudinal path analysis provide evidence for our hypotheses (H1 and H2) and replicate existing research about the outcomes of negative student‐teacher relationships (Roorda et al., 2017) and ethnic discrimination (Benner et al., 2018, 2022; Civitillo et al., 2023), as experiences of unfair treatment from teachers in secondary school were linked with worse academic and socio‐emotional outcomes for adolescents. Going beyond previous studies, which were mainly cross‐sectional, we found that these effects may persist over 5 years and past high school (secondary education). Adolescents who experienced more teacher unfairness at 14 years old (T1) had lower aspirations to go to university 2 years later (controlling for original aspirations at T1), and in turn, were less likely to attend HE (as 18 or 19 years olds) and felt less satisfied with their lives as 19 years olds. As we were able to look at HE enrollment as a hard outcome, our findings show that unfair treatment from teachers can have long‐lasting implications for adolescents' chances in life beyond their school years. In line with Endedijk et al. (2022), we suggest that implementation of strategies that help to reduce negative student–teacher interactions in schools should be one of the crucial efforts in education, beneficial to all students.

#### Effects of perceived teacher unfairness: mediating role of university aspirations and socio‐emotional problems

With our study, we help to unravel the processes through which school social experiences are linked with adolescent adjustment. We found both direct and partially mediated effects of teacher unfairness at T1 on adolescents' outcomes 4‐to‐5 years later (T3 and T4 enrollment in HE and T4 life satisfaction) through the lower aspirations at 16 years (T2). These negative experiences in schools might therefore not only determine adolescents' choices about HE but they may also affect how motivated adolescents feel about continuing their education as 16‐years‐olds, which might have a negative knock‐on effect on subsequent school adjustment—supporting findings about the crucial link between fair school relationships and students' motivation (Chory‐Assad, 2002; Helm et al., 2020; Ripski & Gregory, 2009; Wentzel, 2005). Thus, in schools where we cannot fix the relationships, could we perhaps intervene in other ways to protect student motivation?

Outside the predictable link between university aspirations and university enrollment as two academic outcomes, we also found a link between university aspiration and life satisfaction. The link between decreased university aspirations and other aspects of adolescent school lives and mental health is a path worth investigating in a longitudinal study where researchers control for the initial level of life satisfaction, something that was not possible in our study due to the nature of the secondary dataset.

Another mechanism which we investigated in our study was whether perceived unfairness and discrimination could be linked to university enrolment and life satisfaction through socio‐emotional problems experienced in high school. Thus, addressing H2, we expected that more unfair treatment would be linked to more socio‐emotional outcomes, and in turn worse adjustment at T3 and T4.

Surprisingly, we did not find a direct effect of perceived teacher unfairness at T1 on socio‐emotional problems at T2 when controlling for the initial socio‐emotional problems (at T1). This would imply that over time, teacher unfairness might be less important for socio‐emotional outcomes, unlike our finding about academic outcomes where these effects remained significant over time. We found a significant effect of perceived teacher unfairness on later life satisfaction, yet as mentioned, we were not able to control for the T1 level of life satisfaction. Nevertheless, at the level of descriptive analysis, teacher unfairness was associated with more socio‐emotional problems at T1, suggesting that there could be a link between teacher unfairness and adolescent socio‐emotional functioning. Yet, this effect may not last over time, is not in the direction explored in this study, or is dependent on other moderating or mediating factors such as school‐work related anxiety (Huang, 2020).

#### Effects of perceived ethnic discrimination

In the present study, we acknowledge that “unfair treatment in schools” encompass experiences like being graded harder than other students (labeled here as teacher unfairness) alongside traditionally marginalizing experiences like discrimination, where the unfair treatment is attributed to individuals' ethnic background. In line with our findings about teacher unfairness discussed above, we also found that teacher ethnic discrimination at T1 was associated with a lower likelihood of HE enrollment, the odds ratio suggesting that for every 10 nondiscriminated students enrolled at university, there would be only eight students who experienced discrimination; and this effect was present while we also included control variables such as adolescent ethnicity and SES, and while accounting for the effects of teacher unfairness. However, we did not find any other significant links between perceived teacher or classmate discrimination and longitudinal outcomes measured in our study.

The lack of significant effects in the presence of control variables and teacher unfairness variable is not necessarily contrary to previous findings that perceived ethnic discrimination was associated with worse school and socio‐emotional outcomes (Benner et al., 2018; Cogburn et al., 2011), as the negative correlations between discrimination and T1, T3 andT4 outcomes were all significant but small. Cooper et al. (2022) previously found that perceived teacher discrimination was associated with lower academic aspirations, however, in the referenced study this effect was only significant among boys (not girls). The adverse effects of (in particular peer) discrimination could therefore be conditional on other risk or moderating factors. For example, the link between ethnic discrimination and socio‐emotional outcomes may be more apparent in younger adolescents and in U.S.‐based studies (Benner et al., 2018). Furthermore, these effects might be more pronounced in studies with more detailed measures (Benner et al., 2022; Civitillo et al., 2023). For example, in a study by D'hondt et al. (2016), only frequent experiences of teacher discrimination predicted academic futility, and since our study did not include a measure of discrimination frequency, this might explain why we did not find strong longitudinal effects. The main take‐away from our findings is that teacher negative relationships appear to be more important for school adjustment over time than discrimination by classmates.

Overall, our findings suggest that unfair relationships with teachers in postprimary education have adverse effects on adolescents' school adjustment over time (Benner & Graham, 2011; Cooper et al., 2022) and eventually their life chances at the beginning of their adult lives. These effects are consecutive, as negative relationships at T1 are adversely associated with academic aspirations 2 years later, and in turn both academic and socio‐emotional outcomes in 4 or 5 years. Our findings support the conclusions drawn from other studies that student‐teacher relationships are key for academic outcomes (Roorda et al., 2011). But, they also appear more detrimental to adolescent socio‐emotional outcomes than peer relationships, contrary to previous studies (Benner et al., 2022). Yet, this is possibly through their negative impact on academic outcomes, as school motivation (academic aspirations) was important in mediating the association between T1 teacher unfairness and T3–4 academic and socio‐emotional outcomes (HE enrollment and life satisfaction), more so than the socio‐emotional problems experienced by adolescents at the age of 16. Even though the effects in the current study were generally small, they were significant in the presence of control variables such as adolescent sex or SES and combined, they explain a large proportion of the variance in academic aspirations at 16 years and in HE enrollment.

### Role of historically minoritized ethnic backgrounds

Our study was based on a diverse sample of adolescents from various backgrounds. While we observed that students from all backgrounds could experience unfair treatment in schools, we were particularly interested in the experiences of students from historically minoritized ethnic backgrounds. According to ecological systems theory (Bronfenbrenner, 1977, 1979), social experiences, particularly proximal relationships, form adolescents' developmental competencies, and adjustment. But the close relationships within the microsystem interplay with adolescents' individual differences, as well as sociocultural influences from the macrosystem (García Coll et al., 1996). As such, we investigated the link between adolescents' ethnic background and their school adjustment.

#### School adjustment of historically minoritized adolescents

We did not find that historically minoritized Black or South‐Asian adolescents would be at risk of underachievement in school (Strand, 2010). Instead, adolescents from historically minoritized backgrounds had slightly better academic outcomes than their peers from the White group when controlling for the effects of sex and SES variables as well as the initial T1 outcomes. Both Black‐ and South‐Asian adolescents reported higher university aspirations at T2, and in turn higher likelihood of attending HE (T3 and T4) and slightly better life satisfaction at T4. Additionally, South‐Asian background predicted a higher likelihood of HE enrollment directly, suggesting that these students in particular could have social resources helpful for entering university; perhaps parental influences with an emphasis on achieving high social status (Singh Ghuman, 2002). Future studies could consider aspects omitted from the present study such as the role of family or friends.

In U.S. context, Asian American students are often regarded as model minorities and teachers might in fact hold higher expectation for them (compared with European American students), while the opposite is often the case for other minorities (Tenenbaum & Ruck, 2007). But for South‐Asian youth, the model minority stereotype may be perceived as unfair (Daga & Raval, 2018), and in fact be one of the factors contributing negatively to their mental health (Sharma et al., 2020). Accordingly, in our present study, adolescents from the South‐Asian group also reported slightly more socio‐emotional problems at T2, which had in turn an indirect negative effect on their T4 life satisfaction, effectively canceling out the positive indirect effect of higher aspirations. Furthermore, South‐Asian ethnicity was also associated with lower T4 life satisfaction directly, suggesting that the academic gains could come at the expense of their well‐being. On one hand, our overall finding that historically minoritized adolescents in the UK have better academic motivation (reflected in their aspirations), eventually making them more likely to attend HE, is contributing to the idea of “the immigrant paradox” among minorities in Europe (Dimitrova et al., 2016). On the other hand, they show that due to the contrasting findings among South‐Asian adolescents, further research which measures holistic combinations of psychological, socio‐emotional, and academic aspects is needed.

#### Adolescents ethnic background: moderating and mediating effects

Looking beyond the main effects of ethnic background, we also examined the processes through which students' ethnic background could be connected to adolescent adjustment in postprimary education (RQ2). From a social identity perspective, relationships that have the potential to make adolescents feel socially excluded could be particularly threatening, and as such, historically minoritized adolescents might perceive unfairness and discrimination as a threat (Verkuyten et al., 2019). Accordingly, we investigated whether school unfair treatment would have more adverse effects on school adjustment among minority adolescents than among their majority peers (moderation expectation). A second mechanism that we considered is the *extent* to which adolescents from certain backgrounds might be exposed to unfair treatment, rather than how severely it affects them. Basing our expectation on an integrative model of developmental competencies among children from historically minoritized backgrounds (García Coll et al., 1996), we proposed that certain negative social experiences such as discrimination might be more salient among adolescents from historically minoritized ethnic backgrounds (Huynh & Fuligni, 2010; Verkuyten & Thijs, 2002), and in turn be linked to their developmental processes. Other, more universal processes which are not directly linked to historically minoritized background (such as general teacher unfairness) are relevant for all adolescents, however, we believe that again due to their social position, historically minoritized adolescents might have more of these unfair experiences than their majority peers. As such, we explored whether unfair or discriminatory experiences in secondary school could be the mediators between adolescent ethnic background and their academic and socio‐emotional outcomes (mediation expectation).

##### Moderation model

We did not find strong evidence for the moderation model (i.e., the expectation that unfair treatment would have more prominent effects on outcomes among historically minoritized youth). Specifically, we found only one very small but statistically significant interaction, which, contrary to our expectations, implied that teacher discrimination leads to a slight increase in T2 university aspirations among adolescents from the South‐Asian group, almost as a reactive response to these discrimination experiences. However, this difference in aspirations was negligible and upon further investigation, not statistically significant. Our findings thus suggest that the negative effects of unfair treatment on adolescent academic and socio‐emotional outcomes are similar across different groups.

##### Mediation model

Our findings, while not conclusive, provided more support for the mediation expectation. Minority adolescents experienced more unfair treatment in secondary school than their majority peers, and unfair treatment was linked to more adverse academic and socio‐emotional outcomes. Specifically, adolescents from a historically minoritized background were three to six times more likely to experience ethnic discrimination at T1 (depending on the ethnic group and perpetrator). The effects on unfairness, however, were small and less clear since being from the Black group was only associated with an increase of 0.07 points in the teacher unfairness measure and was associated with a slight decrease (of 0.05 points) in the South‐Asian group. Effects being more pronounced on discrimination is not surprising given that in line with García Coll et al. (1996), experiences of discrimination rise from social stratification and are uniquely linked to minority social status while general teacher unfairness is a more universal experience. The finding that adolescents from a historically minoritized background are significantly more likely to experience unfair treatment by their teachers and peers in school would imply that in turn, these negative experiences in secondary school (at T1) might be associated with more adverse outcomes in the following 2–5 years. However, when tested via mediation, our findings did not provide strong support for the presence of indirect effects. First, none of the specific indirect effects via ethnic discrimination at T1 (by either peers or teachers) were significant at our accepted statistical level (*p* < .01); possibly due to our simplistic measure of discrimination experiences as binary variables due to limitations of using secondary data (discussed below). More complex measures of discrimination which consider aspects such as frequency or severity might be able to capture these indirect effects (if they truly exist) more precisely. Second, while we found that the specific indirect effects via T1 teacher unfairness were statistically significant for three out of four outcomes (bar T2 socio‐emotional problems), these effects were small. Thus, more research is needed to disentangle these somewhat contrasting findings: while adolescents from historically minoritized ethnic backgrounds experience more unfair/discriminatory treatment at T1, and unfair treatment is linked with adverse outcomes over time, the indirect effects from ethnic background to more adverse outcomes via T1 experiences of unfairness/discrimination in schools were small or not significant. We would thus cautiously recommend the mediation expectation as a path worth pursuing in future studies, which should adopt more detailed measures of discrimination.

### Limitations

We utilized a large, diverse, and nationally representative sample of over 13,000 UK adolescents to fill gaps in the current research on the longitudinal effects of unfair treatment experiences with both teachers and peers in postprimary education on school adjustment over the span of 5 years, from mid‐to late adolescence. The use of this dataset carried some limitations, in particular, the lack of standardized or complex measures, which meant some concepts were defined as single‐item measures, which may underestimate the real‐life effects of discrimination (Benner et al., 2022; Civitillo et al., 2023). Nevertheless, single‐item measures of life satisfaction seem to perform just as well as multiple‐item measures (Cheung & Lucas, 2014), and in Benner et al.'s (2022) meta‐analysis, the effect sizes for measures with more items were stronger for socioemotional, but not academic outcomes. Where possible, we attempted to establish validity and reliability of our measures by considering both their theoretical meaning and their structural characteristics (CFA), we would however encourage future studies to replicate our study with more standardized scales. The limitations of the secondary dataset also meant that the variables of interest in the present study were not measured at all time points (i.e., the life satisfaction item was not part of the questionnaire content at T1–T3), and the directionality of these associations can therefore only be presumed based on the presented theory, rather than on testing a fully cross‐lagged model. Furthermore, the data were collected in 2005–2010, and as such it may not fully reflect the experiences of more recent generations of adolescents. We also realize that while aggregating smaller ethnic groups into broader categories allowed us to conduct certain quantitative analyses, it does not fully reflect the diversity among UK adolescents. For example, even our reference group, White adolescents, which is based predominantly on White‐British students has a small proportion of adolescents from other White backgrounds (1.9%); and ethnic or religious discrimination in our study was not exclusively experienced by just those in historically minoritized ethnic categories. Nevertheless, the unique advantage of using such a large dataset based in a European context with objective outcomes (real HE entry rates) and repeated measures, meaning a strong ecological validity of our findings, outweighs some of its limitations.

## CONCLUSIONS

Our study provides an insight into the longitudinal effects of unfair treatment outside of U.S. context. The findings of our present study support the idea that fair treatment and the absence of discrimination in schools promote adolescents' adjustment in school, helping them toward better life chances in adulthood. In particular, perceived teacher unfairness seems to be crucial for adolescent development due to its negative effect on university aspirations and HE enrollment, and life satisfaction over time. Sadly, adolescents from historically minoritized ethnic backgrounds are particularly at risk of experiencing unfair treatment in schools, and as unfair treatment is associated with adverse outcomes, this may be the mechanism through which ethnic background might be linked to their school adjustment over time. Overall, the present study provides a unique insight into the role of perceived unfair and discriminatory treatment in the school microsystem on the lives of UK adolescents. It highlights the importance of promoting teacher fairness for all students, regardless of their ethnic background.

## CONFLICT OF INTEREST STATEMENT

We have no conflict of interest to disclose.

## Supporting information


Data S1.

## Data Availability

This was an existing, publicly available and fully deidentified dataset. The data that support the findings of this study are openly available in UK Data Service, at https://beta.ukdataservice.ac.uk/datacatalogue/series/series?id=2000030#!/access‐data and all input files for our analyses can be downloaded via https://osf.io/eqb5c/?view_only=c150dbf834804f669c1e2b33cfd2ab0a. More details on how the data were collected available in the LSYPE manual.
