# Supplementary material for: The association between experiences of unfair treatment in school and adolescent adjustment over 5 years
Source: J Res Adolesc. 2024 Sep 30;34(4):1545–61. doi: 10.1111/jora.13023 (PMC11606257; doi:10.1111/jora.13023)
Supplement: Supplementary file 1 — Data S1. [file JORA-34-1545-s001.docx]

Supplementary Online Materials

## Ethnic Composition of Aggregated Groups

See Table 1S below for detailed composition of the aggregated ethnic groups used in our study. Please note that ethnic background in the present study refers to both ethnic and racial backgrounds/groups in line with the UK census categories. Participants with missing information on ethnicity, those who self-identified as “Chinese” (n = 36), “Any other mixed background” (n =98) or “Any other” (n = 99) were excluded. Chinese minority group was excluded from the aggregate Asian group since its distinctive school achievement in the UK: 80% of Chinese-background pupils obtained top grade in English and Math, compared to 58% in the aggregate Asian group (Department for Education, 2021).

Table1S Detailed ethnic composition of the aggregated ethnic groups

| Ethnic groups (aggregated) | White | | Asian | | Black | | Total | |
| --- | --- | --- | --- | --- | --- | --- | --- | --- |
|  | *n* | *%* | *n* | *%* | *n* | *%* | *n* | *%* |
| Ethnic origin (self-designation) |  |  |  |  |  |  |  |  |
| White - British | 8964 | 98.1 | 0 | 0.0 | 0 | 0.0 | 8964 | 68.6 |
| White - Irish | 41 | 0.4 | 0 | 0.0 | 0 | 0.0 | 41 | 0.3 |
| Any other White background | 128 | 1.4 | 0 | 0.0 | 0 | 0.0 | 128 | 1.0 |
| White and Black Caribbean | 0 | 0.0 | 0 | 0.0 | 340 | 24.5 | 340 | 2.6 |
| White and Black African | 0 | 0.0 | 0 | 0.0 | 82 | 5.9 | 82 | 0.6 |
| White and Asian | 0 | 0.0 | 150 | 5.9 | 0 | 0.0 | 150 | 1.1 |
| Indian | 0 | 0.0 | 830 | 32.6 | 0 | 0.0 | 830 | 6.4 |
| Pakistani | 0 | 0.0 | 831 | 32.6 | 0 | 0.0 | 831 | 6.4 |
| Bangladeshi | 0 | 0.0 | 623 | 24.5 | 0 | 0.0 | 623 | 4.8 |
| Any other Asian background | 0 | 0.0 | 112 | 4.4 | 0 | 0.0 | 112 | 0.9 |
| Caribbean | 0 | 0.0 | 0 | 0.0 | 453 | 32.7 | 453 | 3.5 |
| African | 0 | 0.0 | 0 | 0.0 | 445 | 32.1 | 445 | 3.4 |
| Any other Black background | 0 | 0.0 | 0 | 0.0 | 66 | 4.8 | 66 | 0.5 |
| Total | 9133 | 100.0 | 2546 | 100.0 | 1386 | 100.0 | 13065 | 100.0 |

## Testing Longitudinal Measurement Invariance in Socio-Emotional Problems

Below, we provide measurement invariance testing for the GHQ-12 as a multiple-item measure which was measured both at T1 and T2. We conducted a series of CFAs to assess (1) configural model, (2) metric invariance, and (3) scalar invariance. We relied on key informal fit indices when comparing model fit, rather than more traditional chi^2^ testing, as chi^2^ tests are sensitive to sample size (Cheung & Rensvold, 2002). To test invariance, we used Chen’s (2007) recommendations, allowing differences of CFI < .01, RMSEA <.015, and SRMR < .030 (for metric) and SRMR < .015 (for scalar). All models had acceptable fit (indicators shown to three decimal places in Table 2S), with comparable values: across models, RMSEA differences were =< 0.001, CFI diff. = 0.001 for metric (vs configural) and 0.009 for scalar (vs metric), SRMR diff. = 0.001 for metric (vs configural) and scalar (vs metric). Furthermore, see Figure 1S for a comparison of the factor loadings from the configural model, in which loadings, intercepts, factor covariance, item residual variance, and residual covariance between the same items at different time points were all freely estimated (while factor means were restricted to 0). See Figure 2S for a comparison of item intercepts from the metric invariance model, in which the factor loadings were set to be equal across time while other parameters were free to vary. Based on this, we conclude that the GHQ-12 indicators were related to the latent factor both at T1 and T2, and these associations were comparable across time.

Figure1S Comparison of GHQ-12 item loadings at T1 and T2 in the configural model


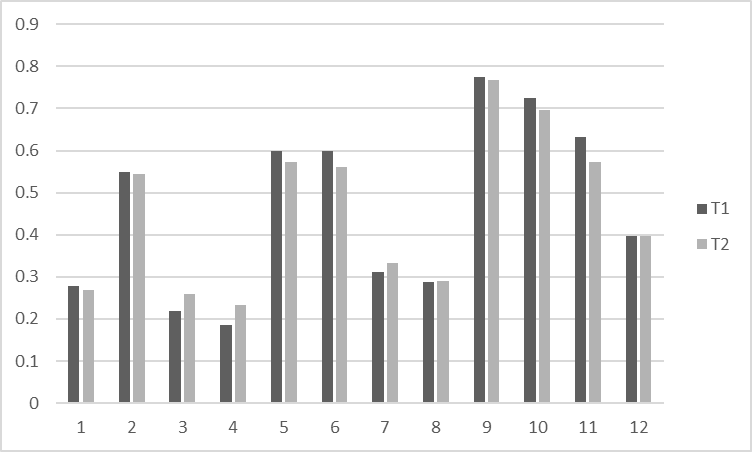


Figure2S Comparison of item intercepts at T1 and T2 in the metric invariance model


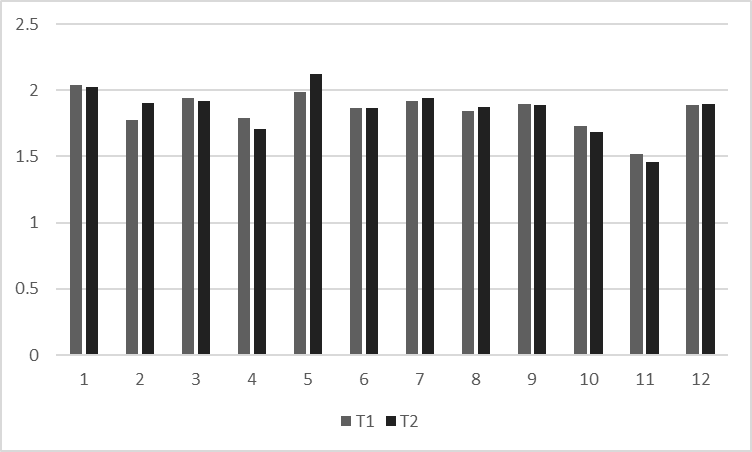


**Table** 2S Comparison of model fit for the configural, metric and scalar invariance models.

|  | **Configural model** | **Metric invariance** | **Scalar invariance** |
| --- | --- | --- | --- |
| df | 239 | 250 | 262 |
| chi^2^ | 8525.33 | 8572.54 | 9127.48 |
| *Scaling correction factor for MLR* | *1.66* | *1.66* | *1.65* |
| RMSEA | 0.052 | 0.051 | 0.051 |
| CFI | 0.857 | 0.856 | 0.847 |
| SRMR | 0.052 | 0.053 | 0.054 |

## Teacher Unfairness: Items and Residual Covariances

We based the teacher unfairness measure on six items. These items were coded on different ordinal scales. We have recoded (i.e., dichotomized) all items, allowing us to combine these into a single measure. This was because unlike the first item (“I get treated unfairly by my teachers”), the other items used were not measuring the extent (but rather just a presence/absence) of unfair treatment. When originally establishing the scale, we have also considered using the following item from the original dataset: “My teachers treat everyone the same regardless of skin colour or cultural background.” After an extensive discussion, we have decided not to include this item into the final scale as it does not focus on perceived unfair treatment (how does the teacher treat *me*) but rather, on fairness as the general characteristic of the teachers in school (how they treat *everyone*). For detailed information about how each item was recoded using SPSS (IBM Corp., 2019)/Mplus 8.3 (Muthén & Muthén, 2019) syntax, see the notes on the dataset section at the end of the supplementary online materials. We observed that with the original items the most common type of response from pupils to the fairness items was that *none* of their teachers treat them unfairly (42% of pupils picked this answer for the question “I get treated unfairly by my teacher”, in contrast only 2% picked that *all* teachers treated them unfairly, 6% that *most*, 18% *some*, 33% *hardly any*) or that they are usually treated the same (in the five other items, the frequencies of choosing *treated* *the same* answer ranged between 63%-76%). This either implies that most students did not experience unfair treatment in post-primary schools, or the questions have not been nuanced enough to distinguish subtler levels of unfair treatment, further supporting our decision to re-code these as binary variables.

We measured the factor structure of the teacher unfairness via a CFA with MLR estimator, and as seen in Table 3S, all items had strong, statistically significant factor loadings, *p* < .001.

Table 3S *Teacher unfairness items and factor loadings (for the recoded-dichotomized items)*

| *Item* | *Original scoring* | *λ* |
| --- | --- | --- |
| I get treated unfairly by my teachers.^i^ | 1 = *All of my teachers*  2 = *Most of my teachers*  3 = *Some of my teachers*  4 = *Hardly any of my teachers*  5 = *None of my teachers* | 0.53^***^ |
| If I get caught breaking school rules then usually I’m… ^ii^ | 1 = *More likely to be punished than others*  2 = *Less likely to be punished than others*  3 = *Treated much the same as anyone else*  4 = *Never break school rules* (recoded as missing) | 0.87^***^ |
| If I’m punished for breaking school rules then usually I get… ^ii^ | 1 = *Punished more heavily than others*  2 = *Punished less heavily than others*  3 = *Treated much the same as anyone else*  4 = *Never break school rules* (recoded as missing) | 0.94^***^ |
| Compared to other pupils in my class my teachers... ^ii^ | 1 = *Are more likely to take an interest in my work*  2 = *Are less likely to take an interest in my work*  3 = *Will treat me much the same as anyone else* | 0.75^***^ |
| Compared to other pupils in my class my teachers… ^ii^ | 1 = *Are more likely to praise my work*  2 = *Are less likely to praise my work*  3 = *Will treat me much the same as anyone else* | 0.74^***^ |
| If there is trouble in a class my teachers… ^ii^ | 1 = *Are more likely to pick on me for causing it than others*  2 = *Are less likely to pick on me for causing it than others*  3 = *Will treat me much the same as anyone else* | 0.80^***^ |

^***^ *p* < .001.

*Note.* ^i^ Dichotomized so that 0 = *none* and 1 = *at least some teachers treat them unfairly*.

^ii^ Dichotomized so *0 = treated less favourable, 1 treated the same or more favourably.*

## Teacher Discrimination

Besides the two questions asked at T1, adolescents were retrospectively asked these questions again at T3, with an implicit instruction to think back to their time at school when they were 12 or 15. We decided to record adolescents as having experienced ethnic discrimination at T1 even when they only indicated this in their retrospective answers. For participants who have not declared that they have experienced discrimination (at T1 and in retrospect), this item was coded as 0. Table 4S presents the four items which were used to create the teacher discrimination measure. If the adolescent responded yes to any of these, this was coded as having experienced teacher discrimination.

Table 4S *Items used for the teacher discrimination measure*

| Items asked at T1: |
| --- |
| Do you think you have ever been treated unfairly by teachers at your school because of your skin colour or ethnic origin? |
| Do you think you have ever been treated unfairly by teachers at your school because of your religion? |
| Items asked at T3 |
| Instruction: *I’d like to ask you a few questions now about when you were in school up to just before you took your GCSEs at the end of Year 11. Thinking back to your time at school when you were 14 or 15…*  Next, still thinking about that same time, do you think you were ever treated unfairly by teachers at your school because of your religion?  Still thinking about that same time, do you think you were ever treated unfairly by teachers at your school because of your race, skin colour or ethnic background? |

*Note*. All items scored as binary (yes/no) items.

## Missingness

We provide a brief overview explaining how we dealt with missing data.

First, in terms of missing data at T1 (which corresponds to wave 2 in the original dataset), the LSYPE study from which our datasets were obtained had generally a good response rate (see Figure 3S): from adolescents sampled for the first wave of data collection, 86 % completed the study. In terms of attrition over the waves (we used W4, W6, W7 as T2, T3, T4 respectively), the responses at repeated waves of data collection (that is, from the second to seventh wave in the original data that corresponds to time 1 to time 4 in our paper) ranged between 86 % - 92 %. The authors of the LSYPE study have ensured that their sample is nationally representative by providing weight variables which take into account the sampling and the missing data, and we have used the weights (from wave 2, i.e., T1 in our manuscript) to ensure that the study sample is nationally representative. More information about the study sampling and weights is available in the LSYPE user guide (Department for Education, 2011).

Figure 3S Response rate in LSYPE


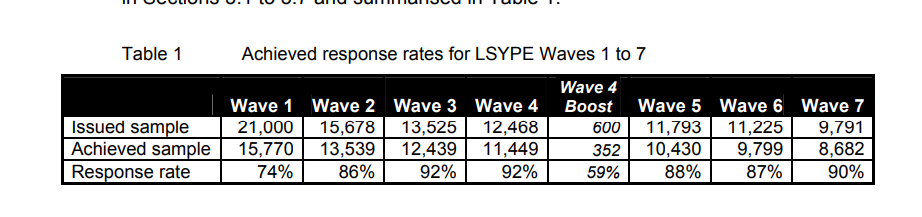
*Note*. From LSYPE user guide [Table], by the Department for Education, 2011

Second, in terms of missingness on specific variables at T1, we were able to estimate these by including covariances between all T1 variables in our models. Missing data were handled using the full-information maximum likelihood (FIML) approach under the missing at random assumption (MAR), that is, missing data can be related to variables in the model. As such, only those with missing on all T1 variables were excluded from our analyses. Similarly, missing data for variables at times 2,3 and 4 were also estimated under FIML approach. FIML uses all available data and it is a preferable approach for handling missing data (Dong & Peng, 2013).

## Moderation & Mediation Expectations: Full model

In Table 5S, we report all effects from the moderation model including those of all the interactions. In Table 6S, we report all direct regression effects from the model testing the expectation that the effect of ethnicity on school adjustment might be mediated via experiences of unfair treatment at T1. Here we report all effects, including the effects of T1 predictors on T2-T4 outcomes, which were omitted from the manuscript, as they were comparable to the findings from the main effects model.

**Table 5S.** *Moderation model: Effects of interaction terms between ethnic group and T1 unfair treatment on school adjustment outcomes*

|  | **T2 University aspirations** | | |  | **T2 GHQ-12** | | |  | **T3-4 Enrolment in HE** | | | |  | **T4 Life satisfaction** | | |
| --- | --- | --- | --- | --- | --- | --- | --- | --- | --- | --- | --- | --- | --- | --- | --- | --- |
|  | *b* | SE | *p* |  | *b* | SE | *p* |  | *b* | SE | *p* | *OR* |  | *b* | SE | *p* |
| *T1 Predictors:* |  |  |  |  |  |  |  |  |  |  |  |  |  |  |  |  |
| Asian | 0.41 | 0.04 | < .001 |  | 0.03 | 0.02 | .150 |  | 0.64 | 0.12 | < .001 | 1.90 |  | -0.12 | 0.05 | .017 |
| Black | 0.38 | 0.05 | < .001 |  | -0.02 | 0.02 | .516 |  | -0.31 | 0.17 | .068 | 0.74 |  | -0.14 | 0.06 | .021 |
| Teacher unfairness | -0.34 | 0.05 | < .001 |  | 0.03 | 0.02 | .182 |  | -1.10 | 0.16 | < .001 | 0.33 |  | -0.18 | 0.05 | .001 |
| Teacher discrimination | -0.10 | 0.04 | .019 |  | 0.02 | 0.02 | .397 |  | -0.40 | 0.13 | .002 | 0.67 |  | 0.03 | 0.05 | .640 |
| Peer discrimination | -0.14 | 0.07 | .046 |  | -0.03 | 0.04 | .399 |  | -0.17 | 0.23 | .459 | 0.85 |  | 0.02 | 0.08 | .821 |
| *T1 Controls:* |  |  |  |  |  |  |  |  |  |  |  |  |  |  |  |  |
| Parental education | 0.09 | 0.01 | < .001 |  | 0.02 | 0.00 | < .001 |  | 0.22 | 0.02 | < .001 | 1.24 |  | 0.01 | 0.01 | .138 |
| HH salary | 0.02 | 0.01 | < .001 |  | 0.00 | 0.00 | .755 |  | 0.08 | 0.02 | < .001 | 1.09 |  | 0.02 | 0.01 | < .001 |
| Sex | 0.12 | 0.02 | < .001 |  | 0.13 | 0.01 | < .001 |  | -0.04 | 0.07 | .525 | 0.96 |  | 0.12 | 0.02 | < .001 |
| *T1 outcomes:* |  |  |  |  |  |  |  |  |  |  |  |  |  |  |  |  |
| T1 University aspirations | 0.65 | 0.01 | < .001 |  |  |  |  |  |  |  |  |  |  |  |  |  |
| T1 GHQ-12 |  |  |  |  | 0.38 | 0.01 | < .001 |  |  |  |  |  |  |  |  |  |
| *T2 outcomes (as mediators):* |  |  |  |  |  |  |  |  |  |  |  |  |  |  |  |  |
| T2 GHQ-12 |  |  |  |  |  |  |  |  | 0.16 | 0.07 | .023 | 1.17 |  | -0.40 | 0.03 | < .001 |
| T2 University aspirations |  |  |  |  |  |  |  |  | 1.53 | 0.04 | < .001 | 4.60 |  | 0.09 | 0.01 | < .001 |
| *Interaction terms:* |  |  |  |  |  |  |  |  |  |  |  |  |  |  |  |  |
| Asian*unfair treatment | 0.19 | 0.12 | .120 |  | 0.09 | 0.06 | .168 |  | 0.30 | 0.36 | .409 | 1.35 |  | 0.09 | 0.13 | .517 |
| Black*unfair treatment | -0.07 | 0.12 | .529 |  | 0.09 | 0.07 | .161 |  | 0.48 | 0.53 | .363 | 1.62 |  | 0.09 | 0.16 | .584 |
| Asian*teacher disc. | 0.19 | 0.07 | .004 |  | 0.09 | 0.04 | .026 |  | 0.35 | 0.22 | .112 | 1.42 |  | 0.03 | 0.08 | .724 |
| Black*teacher disc. | 0.09 | 0.08 | .246 |  | 0.02 | 0.04 | .627 |  | 0.56 | 0.28 | .048 | 1.74 |  | -0.04 | 0.11 | .694 |
| Asian*peer disc. | 0.07 | 0.11 | .537 |  | 0.02 | 0.07 | .741 |  | -0.39 | 0.37 | .286 | 0.68 |  | -0.03 | 0.13 | .803 |
| Black*peer disc. | 0.24 | 0.15 | .126 |  | -0.01 | 0.08 | .901 |  | -0.47 | 0.38 | .220 | 0.63 |  | 0.32 | 0.17 | .053 |
| ***R^2^*** | 0.48 |  |  |  | 0.21 |  |  |  | 0.61 |  |  |  |  | 0.08 |  |  |

**Table 6S** *Regression effects from the ethnicity mediation model with standard errors and odds ratios for binary outcomes*

|  | **T1 Teacher unfairness** | | | **T1 Teacher discrimination** | | | | **T1 Peer discrimination** | | | | **T2 University aspirations** | | | **T2 GHQ-12** | | | **T3-4 Enrolment in HE** | | | | **T4 Life satisfaction** | | |
| --- | --- | --- | --- | --- | --- | --- | --- | --- | --- | --- | --- | --- | --- | --- | --- | --- | --- | --- | --- | --- | --- | --- | --- | --- |
| *T1 Predictors:* | *b* | SE | *p* | *b* | SE | *p* | *OR* | *b* | SE | *p* | *OR* | *b* | SE | *p* | *b* | SE | *p* | *b* | SE | *p* | *OR* | *b* | SE | *p* |
| Asian | -0.05 | 0.01 | < .001 | 1.14 | 0.08 | < .001 | 3.13 | 1.33 | 0.13 | < .001 | 3.77 | 0.50 | 0.03 | < .001 | 0.07 | 0.02 | < .001 | 0.74 | 0.10 | < .001 | 2.09 | -0.10 | 0.04 | .007 |
| Black | 0.07 | 0.01 | < .001 | 1.75 | 0.09 | < .001 | 5.74 | 1.20 | 0.15 | < .001 | 3.32 | 0.39 | 0.04 | < .001 | 0.01 | 0.02 | .500 | -0.06 | 0.12 | .602 | 0.94 | -0.11 | 0.04 | .008 |
| Teacher unfair treatment |  |  |  |  |  |  |  |  |  |  |  | -0.33 | 0.04 | < .001 | 0.04 | 0.02 | .064 | -1.01 | 0.14 | < .001 | 0.36 | -0.16 | 0.05 | .001 |
| Teacher discrimination |  |  |  |  |  |  |  |  |  |  |  | -0.05 | 0.03 | .145 | 0.04 | 0.02 | .034 | -0.22 | 0.10 | .025 | 0.80 | 0.03 | 0.04 | .421 |
| Peer discrimination |  |  |  |  |  |  |  |  |  |  |  | -0.09 | 0.06 | .094 | -0.02 | 0.03 | .405 | -0.31 | 0.17 | .066 | 0.74 | 0.05 | 0.06 | .406 |
| *T1 Controls:* |  |  |  |  |  |  |  |  |  |  |  |  |  |  |  |  |  |  |  |  |  |  |  |  |
| Parental education | -0.01 | 0.00 | < .001 | -0.07 | 0.02 | .002 | 0.93 | 0.03 | 0.04 | .439 | 1.03 | 0.09 | 0.01 | < .001 | 0.02 | 0.00 | < .001 | 0.22 | 0.02 | < .001 | 1.25 | 0.01 | 0.01 | .133 |
| HH salary | -0.01 | 0.00 | < .001 | -0.08 | 0.03 | .004 | 0.92 | -0.16 | 0.04 | < .001 | 0.86 | 0.02 | 0.01 | < .001 | 0.00 | 0.00 | .466 | 0.08 | 0.02 | < .001 | 1.09 | 0.02 | 0.01 | < .001 |
| Sex | -0.05 | 0.01 | < .001 | -0.38 | 0.06 | < .001 | 0.68 | -0.52 | 0.11 | < .001 | 0.60 | 0.12 | 0.02 | < .001 | 0.13 | 0.01 | < .001 | -0.04 | 0.07 | .584 | 0.96 | 0.12 | 0.02 | < .001 |
| *T1 outcomes:* |  |  |  |  |  |  |  |  |  |  |  |  |  |  |  |  |  |  |  |  |  |  |  |  |
| T1 University aspirations |  |  |  |  |  |  |  |  |  |  |  | 0.65 | 0.01 | < .001 |  |  |  |  |  |  |  |  |  |  |
| T1 GHQ-12 |  |  |  |  |  |  |  |  |  |  |  |  |  |  | 0.38 | 0.01 | < .001 |  |  |  |  |  |  |  |
| *T2 outcomes (as mediators):* |  |  |  |  |  |  |  |  |  |  |  |  |  |  |  |  |  | 0.16 | 0.07 | .024 | 1.17 | -0.40 | 0.03 | < .001 |
| T2 GHQ-12 |  |  |  |  |  |  |  |  |  |  |  |  |  |  |  |  |  | 1.53 | 0.04 | < .001 | 4.59 | 0.09 | 0.01 | < .001 |

## Notes on the Data Set

Below is information about the data set. The data in our study is based on the secondary data from the Next steps – originally the Longitudinal study of young people in England. According to the Next steps website, “from 2004 to 2013, Next Steps was run by the UK government’s Department for Education (DfE). In 2013, the management of the study was transferred to the Centre for Longitudinal Studies (CLS) at the UCL Social Research Institute.” The data (University College London, 2021) is publicly available via the UK Data Service^[[1]](#footnote-1)^. We have accessed the non-secure version of the dataset (SN5545) published in 2020. We have merged the datasets from waves two, four, six and seven in SPSS, deleting non-relevant variables. We also shortened the names (in Mplus, variable names cannot exceed eight characters, se the codebook^[[2]](#footnote-2)^), and recoded the variables if required (e.g., all missing values were coded as 999). The shortened version of the dataset was then saved as a csv file and analysed in Mplus 8.3. The waves we have used in our study are as follows:

Wave 2 = Time 1

Wave 4 = Time 2

Wave 6 = Time 3

Wave 7 = Time 4.

### Variable coding

See below the original names of the items which we have used and the syntax used to re-code the relevant variables:

| T1/T2 University aspirations W2uni/W4uni: recoded, based on the original item in W2/W4 dataset heposs9YP |
| --- |
| In SPSS:  *Uni aspirations.  RECODE W2heposs9YP (1=4) (2=3) (3=2) (4=1) (ELSE=999) INTO W2uni.  VARIABLE LABELS W2uni "Likelihood of applying to Uni (recoded W2).".  value labels W2uni  1=not at all likely  2=not very likely  3=fairly likely  4=very likely.  EXECUTE.  *Uni aspirations T2.  RECODE W4Heposs9YP (1=4) (2=3) (3=2) (4=1) (else = SYSMIS) INTO W4uni.  VARIABLE LABELS W4uni 'Likelihood of ever applying to university (recoded W4)'.  Execute.  VALUE LABELS W4uni  1 not at all likely  2 not very likely  3 fairly likely  4 very likely. |
| T1 Teacher unfairness items W2f2_d, W2f3_d, W2f4_d, W2f5_d, W2f6_d : recoded, based on these original items in Wave 2 data set: YYS26 Comp2, Comp3, Comp4, Comp5, Comp6 |
| In SPSS:  RECODE W2YYS26YP (5=1) (4=0) (3=0) (2=0) (1=0) (-999 thru -1=SYSMIS) INTO W2f2_d.  VARIABLE LABELS W2f2_d 'I get treated unfairly by my teachers_dichotomised '.  VALUE LABELS W2f2_d  0 all/most/some/hardly any  1 none.  EXECUTE.  RECODE W2comp2YP (4=999) (2=3) (3=2) (1=1) (-999 thru -1=999) INTO W2f3.  VARIABLE LABELS W2f3 'How likely to get punished for breaking school rules compared to '+  'others_recoded'.  EXECUTE.  RECODE W2comp2YP (4=SYSMIS) (2 thru 3=1) (-999 thru -1=SYSMIS) (1=0) INTO W2f3_d.  VARIABLE LABELS W2f3_d 'How likely to get punished for breaking school rules compared to '+  'others_dichotomised'.  EXECUTE.  RECODE W2comp3YP (4=999) (2=3) (3=2) (1=1) (-999 thru -1=999) INTO W2f4.  VARIABLE LABELS W2f4 'How heavily punished for breaking school rules compared to '+  'others_recoded'.  EXECUTE.  RECODE W2comp3YP (4=SYSMIS) (2 thru 3=1) (-999 thru -1=SYSMIS) (1=0) INTO W2f4_d.  VARIABLE LABELS W2f4_d 'How heavily punished for breaking school rules compared to '+  'others_dichotomised'.  EXECUTE.  RECODE W2comp4YP (1=3) (3=2) ( 2=1) (-999 thru -1=999) INTO W2f5.  VARIABLE LABELS W2f5 'How much interest teacher take in YP work compared to '+  'others_recoded'.  EXECUTE.  RECODE W2comp4YP (1=1) (3=1) (-999 thru -1=SYSMIS) ( 2=0) INTO W2f5_d.  VARIABLE LABELS W2f5_d 'How much interest teacher take in YP work compared to '+  'others_dichotomised'.  EXECUTE.  RECODE W2comp5YP (1=3) (3=2) ( 2=1) (-999 thru -1=999) INTO W2f6.  VARIABLE LABELS W2f6 'How likely to receive praise compared to '+  'others_recoded'.  EXECUTE.  RECODE W2comp5YP (1=1) (3=1) (-999 thru -1=SYSMIS) ( 2=0) INTO W2f6_d.  VARIABLE LABELS W2f6_d 'How likely to receive praise compared to '+  'others_dichotomised'.  EXECUTE.  RECODE W2comp6YP (2=3) (3=2) ( 1=1) (-999 thru -1=999) INTO W2f7.  VARIABLE LABELS W2f7 'How likely teacher to blame YP if there is trouble in class compared with '+  'others_recoded'.  EXECUTE.  RECODE W2comp6YP (2 thru 3=1) (-999 thru -1=SYSMIS) (1=0) INTO W2f7_d.  VARIABLE LABELS W2f7_d 'How likely teacher to blame YP if there is trouble in class compared with '+  'others_dichotomised'.  EXECUTE.  VALUE LABELS W2f3 W2f4 W2f7  1 treated worse_more punished/blame  2 Will treat me much the same as anyone else  3 treated better_less punished/blame.  VALUE LABELS W2f5 W2f6  1 treated worse_less interest/praise  2 Will treat me much the same as anyone else  3 treated better_more interest/praise.  VALUE LABELS W2f3_d W2f4_d W2f5_d W2f6_d W2f7_d  0 treated less favourably  1 Will treat me much the same as anyone else/ more favourably. |
| Parental education W2HQ: was based on two original items from the W2 dataset W2hiqualgmum and W2hiqualgdad. |
| In SPSS:  *SES variables:  parental qualifications.  RECODE W2hiqualgmum (1=7) (2=6) (3=5) (4=4) (5=3) (6=2) (7=1) (else=-999) INTO W2HQmum.  RECODE W2hiqualgdad (1=7) (2=6) (3=5) (4=4) (5=3) (6=2) (7=1) (else=-999) INTO W2HQdad.  VARIABLE LABELS W2HQmum "Mother's highest qualification (recoded).".  VARIABLE LABELS W2HQdad "Father's highest qualification (recoded)".  IF( W2HQmum >= W2HQdad) W2HQ= W2HQmum.  IF( W2HQmum < W2HQdad) W2HQ= W2HQdad.  EXECUTE.  Variable labels W2HQ "Highest qualification parent W2.".  value labels W2HQmum W2HQdad W2HQ  1=No qualification  2=Other qualifications  3=Qualifications at level 1 and below  4=GCSE grades A-C or equiv  5=GCE A Level or equiv  6=Higher education below degree level  7=Degree or equivalent.  EXECUTE. |
| HH salary item salary: based on W2 item Grssyr. |
| In Mplus:  !SALARY  salary = W2Grssyr/10000;  W2sex=W2sex-1;!so 0 Male, 1Female |
| Asian/Black items based on the original item in the W2 dataset ethnicYP. |
| In SPSS:  *new ethnic groups created in the mplus dataset. For m use (-999 thru -1=999).  RECODE W2ethnicYP  (7=999) (15=999) (16=999) (999=999)  (1 thru 3=1)  (6=2) (8 thru 11=2)  (4 thru 5=3) (12 thru 14=3) INTO Eth.  VARIABLE LABELS Eth 'Ethnic groups.'.  EXECUTE.  value labels Eth  1 White  2 Asian  3 Black.  execute.  RECODE Eth (1=0) (3=0) (2=1) (999=999) INTO EthA.  RECODE Eth (1=0) (2=0) (3=1) (999=999) INTO EthB.  execute. |
| T1 Peer discrimination PD: Used items W2praS, based on these items from the original W2 dataset racmotYP, and W2bulrc (if bullied in school). |
| In SPSS:  *racist behaviour peers self report W2racmotYP.  RECODE W2racmotYP (2=0) (3=0.5) (1=1) (else = SYSMIS) INTO W2praS.  VARIABLE LABELS W2praS ' Whether thinks that bullied because peers are racist'.  VALUE LABELS W2praS  0 No  0.5 Sometimes  1 Yes.  EXECUTE.  In Mplus:  !PEER DISCRIMINATION  if (W2praS==0)then PD=0; if (W2bulrc==2) then PD=0;!no racism/never bullied  if (W2praS==1)OR (W2praS==0.5) then PD=1; if W2praP==1 then PD=1; |
| Teacher discrimination TD: Used W2tdis, W2disc1a, W5RaceDi , W5ReliDi based on these original items in Wave 2 data set: Disc1, Disc1a and these original items in Wave 5 data set RaceDis, ReliDis. |
| In SPSS:  *racist behaviour teacher  RECODE W2disc1YP (2=0) (1=1) (else = SYSMIS) into W2tdis.  VARIABLE LABELS W2tdis 'Ever treated unfairly by teachers because of skin colour or ethnic origin.'.  VALUE LABELS W2tdis  0 No  1 Yes.  EXECUTE.  In Mplus:  !TEACHER DISCRIMINATION  if W2tdis==0 then TD=0; if W5RaceDi==2 then TD=0;!no race disc.  if W2disc1a ==2 then TD=0;if W5ReliDi==2 then TD=0;!no relig.disc  if W2tdis==1 then TD=1;if W5RaceDi==1 then TD=1;  if W2disc1a ==1 then TD=1;if W5ReliDi==1 then TD=1; |
| Gender (female) based on W2 dataset item (original name W2SexYP). |
| In Mplus:  W2sex=W2sex-1;!so 0 Male, 1Female |
| T1/T2 GHQL-12 measures GHQL1/GHQL based on the original W2/W4 items as listed below (original SPSS names also contained YP, e.g., W2ConcenYP). |
| !GHQ likert scoring  GHQL1=(mean(W2Concen W2noslep W2Useful W2Decide W2Strain W2Diffic  W2Activ W2Probs W2Depres W2NoConf W2Wthles W2Happy)-1);  GHQL=(mean(W4Concen W4noslep W4Useful W4Decide W4Strain W4Diffic  W4Activ W4Probs W4Depres W4NoConf W4Wthles W4Happy)-1);  !-1 because items scored 1-4, and likert scoring is 0-3 |
| T4 Life satisfaction T4sat. Based on W7 item OSatis (SPSS name W7OSatisYP). |
| In Mplus:  !T4 satisfaction  T4sat=6-W7OSatis;!so 1=v.dissatisfied/5=v.satisfied |
| T3-4 HE enrolment HE: Based on items W6HEFlg, W7HEFlg original names in W6/W7 datasets HEFlag. |
| In Mplus:  !HE  if W6HEFlg==2 then HE=0;if W7HEFlg==2 then HE=0;!not in HE  if W6HEFlg==1 then HE=1; if W7HEFlg==1 then HE=1;!in HE this overwrites the 0 |

# References

Chen, F. F. (2007). Sensitivity of goodness of fit indexes to lack of measurement invariance. Structural Equation Modeling, 14(3), 464–504. [https://doi.org/10.1080/10705510701301834](https://psycnet.apa.org/doi/10.1080/10705510701301834)

Cheung, G. W., & Rensvold, R. B. (2002). Evaluating goodness-of-fit indexes for testing measurement invariance. *Structural Equation Modeling,* *9*(2), 233–255. <https://doi.org/10.1207/S15328007SEM0902_5>

Department for Education. (2011). *LSYPE User Guide to the Datasets: Wave 1 to Wave 7.* UK Data Service. <http://doc.ukdataservice.ac.uk/doc/5545/mrdoc/pdf/lsype_user_guide_wave_1_to_wave_7.pdf>

Dong, Y., & Peng, CY. J. (2013). Principled missing data methods for researchers. *SpringerPlus, 2*, 1-17. <https://doi.org/10.1186/2193-1801-2-222>

Muthén, L. K., & Muthén, B. O. (2019). *Mplus* (Version 8.3) [Computer software]. Muthén & Muthén. https://www.statmodel.com/

Next Steps. (2022, June). *Management and funding.* Centre for Longitudinal Studies, UCL Social Research Institute. https://nextstepsstudy.org.uk/home/about/management-and-funding/

University College London, UCL Institute of Education, Centre for Longitudinal Studies. (2021). *Next Steps: Sweeps 1-8, 2004-2016 (16th ed.)* [Data set]. UK Data Service. https://doi.org/10.5255/UKDA-SN-5545-8

1. <https://beta.ukdataservice.ac.uk/datacatalogue/series/series?id=2000030#!/access-data> [↑](#footnote-ref-1)
2. <https://osf.io/eqb5c/?view_only=c150dbf834804f669c1e2b33cfd2ab0a> [↑](#footnote-ref-2)
